# The clinical management of lenalidomide-based therapy in patients with newly diagnosed multiple myeloma

**DOI:** 10.1007/s00277-020-04023-4

**Published:** 2020-04-16

**Authors:** Maximilian Merz, Tobias Dechow, Mithun Scheytt, Christian Schmidt, Bjoern Hackanson, Stefan Knop

**Affiliations:** 1 Department of Internal Medicine V, Multiple Myeloma Division, Heidelberg University Medical Center, Heidelberg, Germany; 2 Private Oncology Practice Ravensburg, Ravensburg, Germany; 3 Department of Internal Medicine II, Hematology and Medical Oncology Division, Würzburg University Medical Center, Würzburg, Germany; 4 Grosshadern University Hospital, Department of Internal Medicine III, University Hospital of Ludwig Maximilians University, Munich, Germany; 5 Department of Internal Medicine II and ICCA, Augsburg University Hospital, Augsburg, Germany

**Keywords:** Adverse events, Lenalidomide, Multiple myeloma, Newly diagnosed, Safety

## Abstract

Lenalidomide is an integral, yet evolving, part of current treatment pathways for both transplant-eligible and transplant-ineligible patients with newly diagnosed multiple myeloma (NDMM). It is approved in combination with dexamethasone as first-line therapy for transplant-ineligible patients with NDMM, and as maintenance treatment following autologous stem cell transplantation (ASCT). Although strong clinical trial evidence has supported the integration of lenalidomide into current treatment paradigms for NDMM, applying those paradigms to individual patients and determining which patients are most likely to benefit from lenalidomide treatment are more complex. In this paper, we utilize the available clinical trial evidence to provide recommendations for patient selection and lenalidomide dosing in both the first-line setting in patients ineligible for ASCT and the maintenance setting in patients who have undergone ASCT. In addition, we provide guidance on management of those adverse events that are most commonly associated with lenalidomide treatment, and consider the optimal selection and sequencing of next-line agents following long-term frontline or maintenance treatment with lenalidomide.

## Introduction

The treatment of multiple myeloma (MM), a cancer characterized by the clonal expansion of plasma cells [1, 2], has progressed rapidly in recent years. Numerous agents for the treatment of newly diagnosed MM (NDMM) are now available or in development, providing clinicians with a wide array of therapeutic options [3–5]. Whether autologous stem cell transplantation (ASCT) should be used as a first-line strategy in a given subject is a vital clinical decision that must be made soon after diagnosis, and it determines which of two distinct treatment pathways patients will follow [6, 7]. In addition to prolonged progression-free survival (PFS) [8–10], studies have previously shown that patients who undergo ASCT are more likely to achieve a complete response (CR) and minimal residual disease (MRD)-negative status [11] than those who do not, even in the era of novel agents [12, 13]. Although results from the recent FORTE trial showed little difference in response and MRD negativity rates in newly diagnosed patients who do not, versus those who do, undergo transplantation [14], ASCT remains the current first-line standard of care for patients who are able to tolerate this more intensive form of therapy [15]. While stem cells may be collected during first-line treatment and reserved for subsequent ASCT [16], most patients who are eligible for ASCT undergo induction therapy—usually incorporating at least one novel anti-myeloma agent—followed immediately by high-dose therapy and ASCT. Patients may subsequently receive consolidation and/or maintenance therapy. Individuals who are ineligible for ASCT owing to either advanced age or comorbidities follow a different therapeutic path, generally receiving prolonged first-line treatment with a combination of two or more anti-myeloma agents [17, 18].

While most patients ultimately receive multiple lines of therapy during the course of their disease, many do not survive beyond first-line treatment [12]; a deep and durable response to first-line therapy is thus a particularly important treatment goal [19]. The continued development, approval, and validation of increasingly effective combinations of new anti-myeloma agents have increased the likelihood of patients achieving this goal, regardless of the treatment pathway adopted.

The immunomodulatory drug lenalidomide is an integral part of current treatment pathways for both transplant-ineligible and transplant-eligible patients. In newly diagnosed patients not planned to undergo ASCT, lenalidomide plus dexamethasone is approved as a first-line regimen in the United States (US) [20]; in Europe, lenalidomide is indicated for first-line use in combination with dexamethasone with or without bortezomib, and also with melphalan plus prednisone [21]. For patients who do undergo ASCT, single-agent lenalidomide is approved as maintenance treatment post-transplantation [20, 21]; in the US, this applies to any line of treatment [20], whereas the European indication specifies maintenance following first-line ASCT only [21].

This review provides an overview of key studies supporting the approval of lenalidomide in the frontline and maintenance settings. It also presents clinical recommendations for the use of lenalidomide as a component of frontline regimens for patients who are ineligible for ASCT, and as maintenance therapy for patients who have undergone frontline ASCT.

## Key clinical trials of lenalidomide in NDMM

### Lenalidomide as first-line therapy in patients ineligible for, or not scheduled for immediate, transplantation

The approval and use of lenalidomide as a first-line treatment for NDMM patients not undergoing ASCT are supported by two large phase 3 clinical trials, the Frontline Investigation of Revlimid and dexamethasone versus Standard Thalidomide (FIRST) and MM-015 trials, which included patients aged ≥ 65 years or ineligible for ASCT because of comorbidities (Table 1) [22, 23]. In FIRST, lenalidomide plus low-dose dexamethasone (Rd) taken continuously until disease progression significantly prolonged PFS versus either 12 cycles of melphalan, prednisone, and thalidomide (MPT) or eighteen 28-day cycles of Rd (Rd18) [22, 24]. It should be noted, however, that the median duration of actual treatment differed little between the continuous Rd and Rd18 arms in FIRST: 18.4 months and 16.6 months, respectively [22]. Median PFS with Rd until progression was 26 months in FIRST [24], compared with a median PFS of 30 months in patients who received six cycles of Rd in the Southwest Oncology Group (SWOG) S0777 trial [16], and 32 months in patients who received Rd for a median of 21.3 months in MAIA [25]. Continuous Rd also significantly increased overall survival (OS) versus MPT, but not versus Rd18, in FIRST (OS was similar in the continuous Rd and Rd18 groups) [24]. In MM-015, first-line melphalan, prednisone, and lenalidomide (MPR) followed by continuous lenalidomide until progression (MPR-R) significantly prolonged PFS versus either MPR or melphalan plus prednisone (MP) without subsequent lenalidomide (Table 1) [23]. The subsequent phase 3 EMN 01 trial, which compared MPR versus cyclophosphamide, prednisone, and lenalidomide (CPR) versus Rd, reported similar efficacy for MPR versus Rd (Table 1) but a clearly more favorable adverse event (AE) profile for Rd [26], despite melphalan being dose-reduced when compared with the “classic” MP schedule. In the first prospective, randomized, phase 3 trial to apply a frailty-adjusted approach to the treatment of elderly patients with NDMM and an intermediate level of fitness, patients with an International Myeloma Working Group (IMWG) frailty score of 1 were randomized to receive either continuous standard-dose Rd or dose/schedule-adjusted Rd induction followed by lenalidomide maintenance [27] (Table 1). After 25 months’ median follow-up, similar responses, PFS, and OS were observed in the two treatment arms.

**Table 1:** Outcomes in trials evaluating first-line lenalidomide-based regimens in patients ineligible for, or not scheduled for immediate, transplantation

	First-line regimen	PFS, mths	HR (95% CI) for PFS vs. continuous or FD Rd, or MPR-R	OS	HR (95% CI) for OS vs. continuous or FD Rd, or MPR-R
RV-MM-PI-209^a^ [10] Median follow-up: 51 mths	Rd-MPR-R (4 cycles Rd, 6 cycles MPR, continuous R) (*n* = 59)	Median: 34.2		5 years: 70.2%	
	Rd-MPR (4 cycles Rd, 6 cycles MPR) (*n* = 57)	21.8		58.7%	
	Rd-ASCT-R (4 cycles Rd, HDM+ASCT, continuous R) (*n* = 67)	54.7	0.44 (0.32–0.61); *p* < 0.001 for HDM+ASCT±R vs. MPR±R	78.4%	0.55 (0.32–0.93); *p* = 0.02 for HDM+ASCT±R vs. MPR±R at 4 years (81.6% vs. 65.3%)
	Rd-ASCT (4 cycles Rd, HDM+ASCT) (*n* = 68)	37.4		66.6%	
SWOG S0777^a^ [16] Median follow-up: 55 mths	Rd-R, 6 cycles (*n* = 261)	Median^c^: 30	0.71^d^ (0.56–0.91); 1-sided *p* = 0.0018 for Rd-R vs. VRd-R	Median: 64 mths	0.709 (0.52–0.96); 2-sided *p* = 0.0250 for Rd-R vs. VRd-R
	VRd-R, 8 cycles (*n* = 264)	43		75 mths	
FIRST^b^ [22, 24] Median follow-up: 67 mths	Continuous Rd (*n* = 535)	Median: 26.0	0.70 (0.60–0.81)	Median: 59.1 mths	1.02 (0.86–1.20)
	Rd, 18 cycles (*n* = 541)	21.0	0.69 (0.59–0.79); *p* < 0.00001	62.3 mths	0.78 (0.67–0.92); *p* = 0.0023
				49.1 mths	
	MPT, 18 cycles (*n* = 547)	21.9			
MM-015^b^ [23] Median follow-up: 30 mths	MPR-R (9 cycles MPR, continuous R) (*n* = 152)	Median: 31		3 years: 70%	
	MPR, 9 cycles (*n* = 153)	14	0.49; *p* < 0.001	62%	0.79; *p* = 0.25
	MP, 9 cycles (*n* = 154)	13	0.40; *p* < 0.001	66%	0.95; *p* = 0.81
EMN 01^b^ [26] Median follow-up: 39 mths	Rd, 9 cycles (*n* = 217)	Median: 21		4 years: 58%	
	CPR, 9 cycles (*n* = 220)	20	1.01 (0.90–1.13); *p* = 0.938 for CPR vs. Rd	68%	0.93 for CPR vs. Rd
	MPR, 9 cycles (*n* = 217)	24		65%	1.02 for MPR vs. Rd
			0.81 (0.63–1.02); *p* = 0.081 for MPR vs. Rd		
RV-MM-PI-0752^b^ [27] Median follow-up: 25 mths	Continuous Rd (*n* = 101)	20 mths: 42%		20 mths 79%	
	Rd-R (9 cycles Rd, continuous R) (*n* = 98)	43%	0.93 (0.64–1.34); *p* = 0.681	84%	0.73 (0.40–1.33); *p* = 0.306
MAIA^b^ [25] Median follow-up: 28 mths	Continuous Rd (*n* = 369)	Median: 31.9		Median: NR	
	Continuous DRd (*n* = 368)	NR	0.56 (0.43–0.73); *p* < 0.001	NR	Not reported

^a^Patients were not scheduled for immediate transplantation. ^b^Patients were ineligible for transplantation because of age or comorbidities. ^c^Unstratified. ^d^Stratified

*ASCT*, autologous stem cell transplantation; *CI*, confidence interval; *CPR*, cyclophosphamide, prednisone, lenalidomide; *FD*, fixed duration; *HDM*, high-dose melphalan; *HR*, hazard ratio; *mths*, months; *MPR*, melphalan, prednisone, lenalidomide; *MPT*, melphalan, prednisone, thalidomide; *NA*, not available; *NR*, not reached; *OS*, overall survival; *PFS*, progression-free survival; *R*, lenalidomide; *Rd*, lenalidomide plus low-dose dexamethasone

Recent phase 3 trials have shown that the beneficial effects of Rd in the treatment of NDMM can be enhanced by combination with a second novel agent. In the SWOG S0777 phase 3 trial, adding the proteasome inhibitor bortezomib (V) to the Rd doublet backbone (VRd) further prolonged both PFS and OS versus Rd alone as induction therapy in newly diagnosed patients not proceeding to immediate ASCT (Table 1) [16]. Consequently, the VRd triplet is now approved in Europe for use solely in patients deemed ineligible for ASCT [21]. In the phase 3 MAIA trial, the monoclonal antibody daratumumab (D) was added to Rd (DRd), significantly prolonging PFS and increasing MRD negativity and ≥CR rates versus Rd alone (Table 1) [25]. DRd is now also approved in Europe for the treatment of patients with NDMM who are ineligible for ASCT [28].

### Lenalidomide as maintenance therapy following transplantation

Numerous clinical studies have demonstrated the benefits of post-ASCT lenalidomide maintenance treatment in patients with NDMM [10, 29–32]; indeed, two of these studies—Cancer and Leukemia Group B (CALGB) 100104 and Intergroupe Francophone du Myélome (IFM)-2005-02—formed the basis for the approval of lenalidomide in this indication (Table 2) [29, 30]. Both of these studies randomized newly diagnosed patients in remission after ASCT to continuous treatment with lenalidomide or placebo until disease progression [29, 30, 32]. Although 67% of patients in the CALGB 100104 placebo group who did not have progressive disease at this point crossed over to lenalidomide after a median of 18 months’ follow-up, median time to progression (TTP) and OS were significantly longer in the lenalidomide group [30]. Similarly, lenalidomide maintenance significantly increased median PFS versus placebo in IFM-2005-02, although no significant improvement in OS was observed [29]. In order to clarify the effect of lenalidomide treatment on survival outcomes, a meta-analysis was conducted of outcomes in patients who received post-ASCT maintenance in the CALGB 100104, IFM-2005-02, and Gruppo Italiano Malatti Ematologiche dell’Adulto (GIMEMA) RV-MM-PI-209 trials (Table 2) [33]. Of note, patients in the latter were considered on an intent-to-treat basis from the start of first-line therapy. The meta-analysis confirmed a beneficial effect of lenalidomide maintenance on both PFS and OS, despite this not being consistently evident in the individual studies. In addition, both the meta-analysis and the Myeloma XI study showed an improvement in PFS after next therapy (PFS2), indicating that lenalidomide maintenance does not induce progressive disease that is resistant to second-line therapy [31, 33].

**Table 2:** Outcomes following lenalidomide maintenance post-autologous stem cell transplantation

	CALGB 100104 [30, 32] Median follow-up: 91 mths		IFM-2005-02 [29] Median follow-up: 45 mths		Meta-analysis of CALGB 100104, IFM-2005-02, and GIMEMA RV-MM-PI-209 [33] Median follow-up: 79.5 mths		Myeloma XI [31] Median follow-up: 31 mths	
Maintenance therapy	Lenalidomide	Placebo	Lenalidomide	Placebo	Lenalidomide	Placebo or observation	Lenalidomide	Observation
	*n* = 231	*n* = 229	*n* = 307	*n* = 307	*n* = 605	*n* = 603	*n* = 1137	*n* = 834
Median TTP/PFS, mths	TTP: 57.3	TTP: 28.9	PFS: 41	PFS: 23	PFS: 52.8	PFS: 23.5	PFS: 39	PFS: 20
HR (95% CI)	0.57 (0.46–0.71); *p* < 0.0001		0.50; *p* < 0.001		0.48 (0.41–0.55); *p* = NR		0.46 (0.41–0.53); *p* < 0.0001	
OS	Median: 113.8 mths	Median: 84.1 mths	4 years: 73%	4 years: 75%	Median: NR	Median: 86.0 mths	5 years: 61.3%	5 years: 56.6%
HR (95% CI)	0.61 (0.46–0.80); *p* = 0.0004		Not reported; *p* = 0.70		0.75 (0.63–0.90); *p* = 0.001		0.87 (0.73–1.05); *p* = 0.15	

*CI*, confidence interval; *HR*, hazard ratio; *mths*, months; *NR*, not reached; *OS*, overall survival; *PFS*, progression-free survival; *TTP*, time to progression

A recent Belgian study investigated the impact of lenalidomide maintenance on several T cell subsets and myeloid-derived suppressor cells in blood samples from 17 patients with MM who had undergone ASCT [34]. Lenalidomide maintenance was found to increase the proportion of naïve CD8+ and several memory T cell types, while reducing numbers of CD4 and CD8+ terminal effector T cells. The immunosuppressive potential of regulatory T cells became more pronounced with lenalidomide treatment, whereas the activity of myeloid-derived suppressor cells was reduced. The authors concluded that the frequently reported beneficial effects of post-ASCT lenalidomide maintenance on survival outcomes in patients with MM most likely result from a net immunostimulatory effect against residual MM cells.

## Lenalidomide as first-line therapy in NDMM patients ineligible for ASCT: patient selection and dosing

### Patient selection

While lenalidomide is indicated in combination with dexamethasone for the treatment of patients with MM in the US [20], the European label is more specific. For patients with NDMM who are ineligible for ASCT, this approves lenalidomide in combination with the following: dexamethasone until disease progression or unacceptable toxicity; bortezomib and dexamethasone for a maximum of eight 21-day treatment cycles, followed by continued treatment in combination with only dexamethasone; or MP for up to nine 28-day cycles followed by lenalidomide monotherapy [21]. As MM is a highly heterogeneous disease and patient responses to any treatment can vary widely, it is important to determine whether a lenalidomide-based regimen is the best therapeutic option for each individual patient, and to consider how patient- and disease-specific characteristics might impact on the efficacy and feasibility of lenalidomide treatment. Fortunately, results from the pivotal clinical trials described earlier provide some insight into these issues. Real-world data and the results from other clinical trials should also be taken into consideration during clinical decision-making, along with international guidelines and recommendations such as those issued by the US National Comprehensive Cancer Network (NCCN) [15], the European Society for Medical Oncology (ESMO) [35], the IMWG [36], and the European Myeloma Network [37]. Although approved in Europe, MPR-R is not recommended by the NCCN or ESMO for the treatment of transplant-ineligible patients; therefore, this review focuses principally on the more widely used and recommended Rd.

Bone marrow function is an important consideration when selecting the initial treatment regimen for ASCT-ineligible patients. According to the European label, absolute neutrophil count (ANC) must be ≥ 1.0 × 10^9^/L and/or platelet count ≥ 50 × 10^9^/L prior to commencing treatment with Rd [21]. Renal impairment must also be considered prior to starting treatment. As lenalidomide is excreted renally, patients with impaired renal function may require dose adjustments (see below) that could impact on treatment efficacy; thus, depending on the severity of renal impairment and the level of dose adjustment required, alternative regimens may be more appropriate options. In the FIRST trial, patients with mild-to-moderate renal impairment (creatinine clearance [CrCl] ≥ 30 to < 80 mL/min) who received continuous Rd obtained a similar PFS benefit versus MPT to those with normal renal function, despite dose adjustments; these benefits were coupled with improved renal function in the majority of patients with IMWG-defined complete renal response (CR^renal^; this was achieved by 23.8% and 14.3% of Rd and MPT recipients, respectively) [38]. The observed improvement in renal function may have resulted from elimination of the toxic free light chains that are associated with MM. However, no benefit of Rd over MPT was noted in patients with severe renal impairment (CrCl < 30 mL/min), suggesting that Rd may not be the optimal treatment choice for this patient population. Based on IMWG recommendations, bortezomib-based regimens should be considered for such patients [39].

Sub-analyses of the pivotal clinical trial data in patients ineligible for ASCT suggest that the survival benefit seen with lenalidomide-based regimens versus comparators is largely independent of many patient- and disease-related factors, including gender, ethnicity, International Staging System (ISS) stage, renal function, Eastern Cooperative Oncology Group (ECOG) performance status, and β_2_-microglobulin level [22, 40]. In the FIRST trial, continuous Rd was more effective in prolonging survival than MPT, regardless of whether patients were older or younger than 75 years [22, 40]. However, long-term data from this trial suggested that continuous Rd offered no survival benefit versus MPT in patients with high-risk cytogenetics or high lactate dehydrogenase (LDH) levels at diagnosis [24]. These latter findings are in contrast to those from the SWOG S0777 trial, which reported a greater beneficial effect of VRd versus Rd in patients with high-risk cytogenetics than in the overall population [16]. A pooled analysis of data from the GIMEMA MM-03-05 and EMN 01 trials also found that, in patients with high-risk cytogenetics, treatment with a bortezomib-based regimen significantly prolonged both PFS and OS versus lenalidomide-based treatment [41]. These data suggest that adding a proteasome inhibitor to Rd may help overcome the adverse prognostic impact of high-risk cytogenetics, and is consistent with the IMWG recommendation that NDMM patients with high-risk cytogenetics receive combination therapy with a proteasome inhibitor, lenalidomide or pomalidomide, and dexamethasone [42].

When selecting a first-line therapy for NDMM patients ineligible for ASCT, physicians should carry out a full work-up, including tests for cytogenetics, renal function, hematologic function, and venous thromboembolism risk, to enable them to assess the suitability of a lenalidomide-based regimen, and whether Rd is the most appropriate treatment or whether bortezomib should be added. Guidance on managing the toxicities most commonly encountered with lenalidomide-based regimens is provided later in this review.

### Dosing and treatment duration

When used in combination with dexamethasone, lenalidomide is administered at a dosage of 25 mg once daily on days 1–21 of 28-day cycles [20, 21]. Dexamethasone dosing is normally 40 mg once daily on days 1, 8, 15, and 22 of each 28-day cycle, although the starting dose may be reduced to 20 mg once daily in patients aged > 75 years [20, 21]. Dexamethasone dose reduction was successfully demonstrated in the previously described trial investigating a frailty-adjusted approach in elderly patients with intermediate levels of fitness [27]. In that trial, treatment with 9 cycles of lenalidomide in combination with dexamethasone 20 mg once weekly, followed by lenalidomide maintenance until progression, was associated with response rates and survival outcomes that were comparable with those seen with continuous standard-dose Rd. Rates of non-hematologic grade 3/4 AEs, and lenalidomide dose reductions and discontinuations, were reduced with the modified Rd-R regimen versus continuous standard-dose Rd, and the lenalidomide median relative dose intensity was 100% versus 90%. On the basis of these findings, pre-emptive dexamethasone dose reductions should be considered in frail and/or elderly patients as a means of reducing the risk of toxicities that could negatively impact treatment adherence and outcomes in such populations. The starting dose of lenalidomide may also be reduced in elderly patients; however, this is not in accordance with the approved use, and studies to confirm comparable efficacy are needed. Administration of both lenalidomide and dexamethasone should be continued until either disease progression or intolerance [20, 21].

When combined with bortezomib and dexamethasone, the lenalidomide dosage is 25 mg once daily on days 1–14 of 21-day cycles, with bortezomib administered subcutaneously at a dosage of 1.3 mg/m^2^ on days 1, 4, 8, and 11 of each cycle (a recent review and meta-analysis confirmed similar survival and response rates with subcutaneous [sc] versus intravenous bortezomib, but significantly reduced rates of adverse events such as peripheral sensory neuropathy, fatigue, and thrombocytopenia [43]. As bortezomib was administered intravenously in SWOG-0777 [15], switching to sc administration would be expected to lead to noticeably lower bortezomib-associated neurologic toxicity rates than were reported in that trial). This regimen is recommended for a maximum of eight cycles before bortezomib is omitted and treatment continued with lenalidomide (25 mg once daily on days 1–21) plus dexamethasone in 28-day cycles until either disease progression or unacceptable toxicity [21].

Lenalidomide dose adjustments are required for patients with renal impairment, regardless of which regimen is used. The recommended dose in patients with moderate renal impairment is 10 mg once daily; if the patient is able to tolerate it, this may be increased to 15 mg if he or she does not respond to treatment with 10 mg once daily [20, 21]. Patients with severe renal impairment not requiring dialysis should receive 15 mg every second day or 7.5 mg daily if this latter capsule size is available locally. Individuals with severe renal impairment requiring dialysis should receive 5 mg once daily; on dialysis days, lenalidomide should be administered after dialysis.

It is important for patients to remain on lenalidomide treatment for as long as possible; this is underscored by the FIRST study, in which both PFS and time to next treatment (TTNT) were prolonged in patients receiving continuous Rd versus those assigned to Rd18 (median PFS, 26.0 versus 21.0 months, respectively; median TTNT, 36.7 versus 28.5 months) [22, 24]. Dosing should be adjusted for toxicity, and any problems addressed as quickly and effectively as possible in order to enable patients to continue with the prescribed regimen and achieve the maximal benefit.

## Use of lenalidomide as maintenance therapy

### Patient selection

In both the US and Europe, lenalidomide is recommended as maintenance therapy following ASCT in patients with NDMM [15, 35]. As with first-line use in patients ineligible for ASCT, it is important to consider an individual patient’s likelihood of benefiting from maintenance therapy before making treatment recommendations. Single-agent lenalidomide maintenance, like Rd, can be given until progression; thus, patients at increased risk of potential complications owing to long-term lenalidomide exposure should be identified in advance (this will be discussed later in the toxicity section). In addition to the clinical trial data described earlier, findings from real-world studies [44–46] and additional clinical trials [31] may be valuable in this respect. Although the pivotal studies suggest that many patients will benefit from maintenance treatment with lenalidomide, these results must be interpreted with caution. The findings from the pivotal studies and the associated meta-analysis show an overall benefit of lenalidomide maintenance, albeit with some heterogeneity between individual studies and subgroups; thus, individual treatment decisions should be made on a case-by-case basis.

In view of the known hematologic toxicity of lenalidomide, patients with compromised bone marrow function following ASCT may not be suitable for maintenance therapy and/or may require a delay in treatment until their hematologic function has recovered to an adequate level. According to both the US prescribing information and the European summary of product characteristics, lenalidomide maintenance post-ASCT should only be initiated when ANC is ≥ 1.0 × 10^9^/L and/or platelet count ≥ 75 × 10^9^/L [20, 21]. Additionally, lenalidomide should not be administered to patients with a history of severe rash associated with thalidomide treatment [20, 21].

It is not yet clear whether patients can be selected for lenalidomide maintenance on the basis of individual patient- and disease-specific characteristics. A number of studies have analyzed the impact of disease stage on survival, with no clear consensus [10, 31, 33]. The largest of these was the meta-analysis discussed above, which suggested that patients with ISS stage I or II disease may derive more benefit from lenalidomide maintenance than those with stage III disease [33]. Individual studies suggest that lenalidomide maintenance may be equally advantageous for patients of all ages [10, 29, 31], although the results of the meta-analysis indicated a reduced survival benefit in patients aged > 60 years [33]. While relevant data were not available for all patients, the same analysis found that lenalidomide maintenance provided no OS benefit in patients with elevated LDH levels or CrCl < 50 mL/min at diagnosis [33], suggesting that its value may be limited in such populations. Similarly, the benefits and risks of lenalidomide in patients with impaired hepatic function should be carefully evaluated, as these patients may be at risk of potentially fatal hepatotoxicity [20].

As is the case for definitive first-line therapy, the relative benefit of lenalidomide maintenance versus no maintenance is limited in patients with high-risk cytogenetics, but is typically much greater in patients with standard-risk cytogenetics [10, 33]. Nevertheless, some studies suggest that lenalidomide maintenance significantly prolongs survival and/or TTNT versus no maintenance in patients considered at high cytogenetic risk [31, 46]. In the Myeloma XI trial, lenalidomide maintenance prolonged both PFS and OS versus observation in all cytogenetic risk groups (standard, high, and ultra-high) [31]. Three-year OS with lenalidomide maintenance versus observation was 74.9% versus 63.7% among high-risk patients, and 62.9% versus 43.5% among those considered to be at ultra-high risk. However, as these subgroup analyses were not adequately powered for direct comparison, the authors advise that their findings are inconclusive and must be interpreted with caution.

As well as varying with cytogenetic risk profile, the benefits of lenalidomide maintenance may differ according to the depth of response to ASCT, with patients who achieve deeper responses (≥ very good partial response [VGPR]) appearing to derive greater benefit than those with a lesser response [33]. Taken together, these findings suggest that alternatives to lenalidomide maintenance (for example, bortezomib administered for 2 years [47]), or even no maintenance treatment, may be preferable in patients with high-risk cytogenetics or with a lesser response to ASCT. All other patients may benefit from lenalidomide maintenance, and suitability should be assessed on a case-by-case basis.

### Dosing

When given as maintenance treatment, the recommended starting dose of lenalidomide is 10 mg once daily on days 1–28 of repeated 28-day cycles, until either disease progression or unacceptable toxicity [20, 21]. If tolerated, the dose may be increased to 15 mg/day after three cycles of lenalidomide maintenance [20, 21], consistent with the dosing regimen used in pivotal clinical trials [29, 30]. In routine clinical practice, prolonged treatment at these dosages may be achievable in only a proportion of patients; however, real-world data suggest that dose reductions do not adversely impact PFS [48]. The US prescribing information recommends that patients with moderate renal impairment (CrCl 30–60 mL/min) receive 5 mg once daily, while those with severe renal impairment (CrCl < 30 mL/min), regardless of whether dialysis is required, receive 2.5 mg once daily. In Europe, the recommendation is 10 mg once daily in patients with 30 ≤ CrCl < 50 mL/min, 7.5 mg once daily or 15 mg every second day in patients with severe renal impairment not requiring dialysis, and 5 mg once daily in patients with end-stage renal disease requiring dialysis [20]. In patients undergoing dialysis, lenalidomide should always be administered after dialysis [20, 21].

### Determining the optimal duration of lenalidomide maintenance treatment

In most studies, including the pivotal phase 3 trials, it was intended that lenalidomide should be administered until the development of either disease progression or unacceptable toxicity [10, 29–31, 33]; in practice, however, the actual duration of maintenance treatment has varied. In the IFM 2005-02 trial, the median duration of lenalidomide maintenance administration was approximately 2 years, while in the CALGB 100104 and GIMEMA RV-MM-PI-209 trials, it was 2.5 and 3 years, respectively [33]. Whether fixed-duration maintenance results in improved outcomes versus treatment until progression is currently unclear. A retrospective analysis of 464 patients who received lenalidomide maintenance post-ASCT suggested an association between longer duration of maintenance therapy and improved PFS and OS; however, this particular analysis was limited by its retrospective nature, in addition to missing data and a lack of standardization for second primary malignancy (SPM) screening [49]. In the phase 3 GMMG-MM5 study, patients received one of two bortezomib-based induction regimens followed by ASCT, as well as lenalidomide consolidation and maintenance for either 2 years or until the achievement of CR [50]. OS was significantly longer in 2-year lenalidomide recipients than in those who received maintenance only until CR (hazard ratio [HR] 1.42 [95% CI, 1.04–1.93]; *p* = 0.03). Interestingly, only 35% of patients assigned to 2 years of maintenance actually received treatment for this length of time; even fewer patients in the treatment until CR arms (14–18%) received 2 full years of treatment. Whether continued maintenance can achieve further cytoreduction and potentially even eliminate residual myeloma cells remains to be seen. Thus, based on the available data, lenalidomide maintenance should be given for as long as possible.

## Management of hematologic AEs

### Neutropenia

Neutropenia is the most common and most significant lenalidomide-associated AE, and occurs most frequently at grade 3 or 4 (Tables 3 and 4). Grade 3/4 neutropenia was reported in 30% of patients receiving first-line continuous Rd in the FIRST trial [24], and high rates have been reported with long-term maintenance also: 51% and 50% in lenalidomide-treated patients in IFM-2005-02 and CALGB 100104, respectively [29, 30]. Like most hematologic AEs seen with lenalidomide, neutropenia occurs most frequently during the first 6 months of treatment, with incidences declining over time [22], and can be managed by interrupting treatment, monitoring complete blood count, and waiting until toxicities resolve before starting the next treatment cycle [51]. Physicians should carry out a complete blood cell count—including white blood cell count with differential count, platelet count, hemoglobin, and hematocrit—at baseline, weekly for the first 8 weeks, and then monthly thereafter [21]. Patients with neutropenia should be monitored for signs of infection [52]. If hematologic toxicity continues, stepwise reductions of the lenalidomide dose should be implemented according to the prescribing information or summary of product characteristics, and growth factor support should be considered if neutropenia is severe and/or the only toxicity observed [20]. In patients receiving first-line treatment, lenalidomide may be re-introduced at the next higher dose level, up to the starting dose, upon improvement of bone marrow function, as indicated by a lack of hematologic toxicity for at least two consecutive cycles (i.e., ANC ≥ 1.5 × 10^9^/L with platelet count ≥ 100 × 10^9^/L at the beginning of a new cycle) [21]. Patients should be advised to promptly report febrile episodes. Rates of grade 3/4 febrile neutropenia in the FIRST trial were 1% with continuous Rd and 3% with Rd18 [24], while rates of up to 17% have been reported in maintenance studies [20].

**Table 3:** Incidence of selected grade ≥ 3 AEs and SPMs in key clinical trials of first-line lenalidomide-based regimens in patients ineligible, or not scheduled, for transplantation

AE, %													
	First-line regimen	Neutropenia	Anemia	Thrombocytopenia	Fatigue	Diarrhea	Constipation	Infection	Cardiac disorder	Dermatologic AE	DVT	PE	SPM
FIRST [22, 24 Median follow-up: 67 months	Continuous Rd (*n* = 532)	30	19	9	NR	5	2	32	NR	NR	5	4	7
	Rd, 18 cycles (*n* = 540)	26	16	8	NR	3	2	22	NR	NR	4	3	7
	MPT, 18 cycles (*n* = 541)	45	19	11	NR	1	5	17	NR	NR	3	4	9
MM-015^a^ [23] Median follow-up: 30 months	MPR-R (9 cycles MPR, continuous R) (*n* = 150)	67/35	24/3	35/11	5/0	2/1	NR	9/1	3/2	5/0^b^	1/0	NR	3-year risk: 7
	MPR, 9 cycles (*n* = 152)	64/32	26/3	38/12	1/1	1/0	NR	13/2	3/3	5/0^b^	4/1	NR	7
	MP, 9 cycles (*n* = 153)	29/8	14/1	12/4	3/0	0/0	NR	7/0	3/0	1/0^b^	1/0	NR	3
EMN 01^a^ [26] Median follow-up: 39 months	Rd, 9 cycles (*n* = 212)	25	4	7	2	NR	NR	9	6	5	2^c^	NR	0
	CPR, 9 cycles (*n* = 220)	29	6	9	2	NR	NR	7	6	8	5^c^	NR	1
	MPR, 9 cycles (*n* = 211)	64	15	18	3	NR	NR	11	5	5	3^c^	NR	2
RV-MM-PI-0752 [27] Median follow-up: 25 months	Continuous Rd (*n* = 101)	14	NR	NR	NR	NR	NR	11	NR	7^d^	NR	NR	NR
	Rd-R (9 cycles Rd, continuous R) (*n* = 98)	17	NR	NR	NR	NR	NR	9	NR	3^d^	NR	NR	NR
MAIA [25] Median follow-up: 28 months	Continuous Rd (*n* = 369)	35	20	NR	4	4	0	23	NR	NR	NR	NR	7
	Continuous DRd (*n* = 368)	50	12	NR	8	7	2	32	NR	NR	NR	NR	8

^a^Events reported during induction. ^b^Grade 3/4. ^c^Incidence of DVT/TE. ^d^Incidence of rash

*AE,* adverse event; *CPR*, cyclophosphamide, prednisone, lenalidomide; *DVT*, deep-vein thrombosis; *FD*, fixed duration; *HDM*, high-dose melphalan; *MPR*, melphalan, prednisone, lenalidomide; *MPR-R*, melphalan, prednisone, lenalidomide + continuous lenalidomide until progression; *MPT*, melphalan, prednisone, thalidomide; *NR*, not reported; *PE*, pulmonary embolism; *Rd*, lenalidomide plus low-dose dexamethasone; *SPM*, second primary malignancy; *TE*, thromboembolism

**Table 4:** Incidence of selected grade ≥ 3adverse events and second primary malignancies in key clinical trials of lenalidomide maintenance therapy post-autologous stem cell transplantation

	CALGB 100104 [30] Median follow-up: 91 months			IFM-2005-02 [29] Median follow-up: 45 months		Myeloma XI [31] Median follow-up: 31 months	
	Lenalidomide^a^	Placebo (no crossover)^a^	Placebo (crossover)^a,b^	Lenalidomide	Placebo	Lenalidomide^a^	Observation
	*n* = 231	*n* = 143	*n* = 86	*n* = 306	*n* = 302	*n* = 1137	*n* = 834
Neutropenia	35/15	5/3	30/5	51	18	28/5	NR
Anemia	4/1	0/0	1/0	3	2	4/1	NR
Thrombocytopenia	10/5	0/5	3/2	14	7	4/2	NR
Fatigue/lethargy	0/0	0/0	0/0	5	2	1/0	NR
Diarrhea	5/0	1/0	3/0	2	< 1	NR	NR
Constipation	NR	NR	NR	1	0	< 1/0	NR
Infection	6/1	2/0	5/0	13	5	11/1	NR
Cardiac disorder	NR	NR	NR	NR	NR	0/0	NR
Rash	4/0	1/0	1/0	3	2	1/< 1	NR
Thromboembolic event	NR	NR	NR	2/1^c^	1/0^c^	NR	NR
Second primary malignancy^d^, %	19	3	15	10	4	5/3^e^	NR

^a^Grade 3/4. ^b^Patients who crossed over to lenalidomide treatment during the trial. ^c^Deep-vein thrombosis/pulmonary embolism. ^d^Not graded. ^e^3-year cumulative incidence

*NR*, not reported

### Thrombocytopenia

Grade 3/4 thrombocytopenia was reported in 9% of patients receiving first-line continuous Rd in FIRST [24], while the incidence of such events in patients receiving post-ASCT lenalidomide maintenance in the IFM-2005-02 and CALGB 100104 studies was 14% and 15%, respectively (Tables 3 and 4) [29, 30]. Patients and physicians should watch for signs and symptoms of bleeding, including petechiae and epistaxes, especially in patients treated concomitantly with medicines that may induce bleeding [21]. Complete blood counts should be taken weekly, particularly during the first few cycles, and the dose interrupted if platelet levels fall to < 30,000/mm^3^ [52]. Once platelet levels have recovered, treatment should be resumed at the next lower dose. For each subsequent drop to < 30,000/mm^3^, treatment should be interrupted and resumed at the next lowest dose upon recovery. Platelet transfusions according to standard clinical practice may be necessary. There are currently no recommendations supporting the use of thrombopoietic agents to manage thrombocytopenia in patients receiving lenalidomide treatment.

### Anemia

Anemia is most common among patients treated with first-line lenalidomide-containing regimens, with grade 3 or 4 events affecting 19% of those treated with continuous Rd in FIRST [24]. By contrast, the incidence of grade 3/4 anemia was 3–5% in patients receiving lenalidomide maintenance in IFM-2005-02 and CALGB 100104 (Table 4) [29, 30]. As for thrombocytopenia, the incidence of anemia appears relatively consistent over time, occurring only slightly more frequently during the first 6 months of treatment [22]. Although erythropoiesis-stimulating agents (ESAs) are recommended for MM patients with persistent symptomatic anemia [53], these agents should be used with caution, as they may increase the risk of thromboembolism when co-administered with lenalidomide and dexamethasone [54]. Otherwise, anemia should be managed according to standard clinical practice, and severe cases may require transfusions.

### Thromboembolism

Thromboembolism is a serious lenalidomide-associated AE that occurs most frequently during first-line treatment, as the risk is elevated when co-administered with dexamethasone (Tables 3 and 4) [22, 23, 29, 32, 55]. Despite thromboprophylaxis with either low-dose aspirin or a low-molecular-weight heparin, heparin, or warfarin, 5% of patients treated with continuous Rd in the FIRST trial experienced deep-vein thrombosis (DVT), and 4% developed a pulmonary embolism (PE) [24]. When given as maintenance therapy following ASCT, thromboembolic events rarely occur with lenalidomide treatment if co-administered with judicious anticoagulation [29, 32]. No patients were affected in the CALGB 100104 trial, in which all those considered to be at high risk of DVT or PE received aspirin, warfarin, or heparin [32]; however, in the IFM-2005-02 trial, in which thromboprophylaxis was not used, 6% of lenalidomide-treated patients developed any-grade DVT or PE [29]. Although thromboembolism may occur at any time, it usually occurs more frequently in the first months of lenalidomide therapy; hence, physicians should monitor patients particularly closely during the early stages of therapy [22, 55, 56].

Prophylactic antithrombotic medications should be recommended to all patients taking lenalidomide, especially those with additional thrombotic risk factors [21, 57]. Based on clinical trial data in patients receiving first-line lenalidomide-based regimens, oral anticoagulation with low-dose aspirin may be an effective and less expensive alternative to low-molecular-weight heparin (LMWH) prophylaxis in patients with low thromboembolic risk [56]. Recently published data for apixaban, a new direct factor Xa inhibitor, suggest that agents of this type may also constitute a safe and convenient thromboprophylactic alternative to LMWHs in this indication [58]. Aspirin or a LMWH should, unless contraindicated, be administered to patients receiving epoietin therapy concomitantly with lenalidomide maintenance [32], and patients with known risk factors for thromboembolism, including prior thrombosis, should be closely monitored and all modifiable risk factors minimized [21]. Medications that may increase the risk of thrombosis, such as hormone replacement therapy, should be used with caution in patients receiving Rd [20]. Patients and physicians should be alert to the signs of thromboembolism, including shortness of breath, chest pain, or arm or leg edema [20, 21]. If any thromboembolic events occur, treatment should be discontinued and anticoagulation therapy commenced; lenalidomide treatment may be restarted, along with appropriate antithrombotic therapy, once the thromboembolic event has been treated, and after a careful risk-benefit assessment [21].

## Management of non-hematologic AEs

Physicians should be alert for non-hematologic AEs and manage any that arise as promptly and effectively as possible, in order to facilitate optimal dosing, minimize treatment disruptions and discomfort to patients, and maximize treatment duration and benefit [52, 59]. Patients and caregivers should be informed of the potential for adverse reactions and their possible symptoms and manifestations, and be encouraged to report any early signs. Peripheral neuropathy, which can be problematic with other novel agents such as bortezomib and thalidomide [60–62], is fortunately seen far less frequently in patients treated with lenalidomide [20, 22, 29, 32]. Described below are some of the most common non-hematologic adverse reactions seen with lenalidomide treatment. Patients receiving Rd may also experience dexamethasone-related AEs, such as hyperglycemia and psychological changes; if suspected, the dexamethasone dose may be reduced [63].

### Rash

Rash is a very common side effect of lenalidomide treatment [21]. These skin reactions are generally mild-to-moderate in severity; however, rash of higher grades may occur (Tables 3 and 4) [29, 30, 59]. Lenalidomide-induced rash may present as patchy, raised, macular skin lesions, sometimes with localized urticaria [59], and is more likely to occur during the first cycle of therapy [64]. All patients should be routinely monitored for skin-related AEs during treatment [65]. Mild-to-moderate rash can be treated with antihistamines, topical corticosteroids, and/or appropriate dose modifications [59, 64, 65]. Treatment interruption or discontinuation should be considered for grade 2 or 3 skin rash, and lenalidomide must be discontinued for angioedema, grade 4 rash, exfoliative or bullous rash, or if serious reactions, including Stevens-Johnson syndrome, toxic epidermal necrolysis, or Drug Reaction with Eosinophilia and Systemic Symptoms, are suspected [21]. Patients should be informed of the signs of these serious reactions and advised to seek medical attention immediately if they develop any symptoms. Treatment should not be resumed after discontinuation owing to any of these events [20].

### Diarrhea and constipation

Diarrhea occurs frequently in patients treated with lenalidomide (Tables 3 and 4), although most commonly at lower grades [30], and may manifest as late as 17–24 months after treatment initiation [66]. If poorly managed, diarrhea can lead to multiple physiologic abnormalities, reduce patients’ quality of life, and lead to unnecessary treatment discontinuation [66, 67]; thus, proactive management is essential. Symptomatic treatment of diarrhea with medications such as loperamide or colesevelam, coupled with adequate hydration, is appropriate for most patients [52, 68]; however, as research suggests that lenalidomide-induced diarrhea may be related to bile acid malabsorption (BAM), investigations for this condition should be carried out if practical, and a trial of bile acid sequestrant therapy, along with dietary adjustments, initiated [52, 67]. Bearing in mind that lenalidomide capsules contain lactose, intolerance to lactose should also be considered [52].

Constipation is another common occurrence with lenalidomide treatment (Tables 3 and 4) and, along with the associated bowel discomfort, can be a disabling toxicity [68]. Lenalidomide-related constipation can be managed with general supportive care measures, such as increased fluid and fiber intake, pharmacological agents, increased physical activity, and laxatives or stimulants. Assessment for bowel obstruction, intravenous hydration, disimpaction, and referral to a gastroenterologist may be required for severe cases [65, 68]. Dose interruptions/modifications may also be necessary [65].

### Fatigue

Fatigue of grade 3 or higher has been reported in up to 18% of lenalidomide-treated patients [69], and is a common reason for treatment discontinuation [64]. While fatigue is common in all patients with NDMM [65, 70], lenalidomide-treated patients may experience more frequent and severe fatigue than patients receiving placebo (Tables 3 and 4) [29] or bortezomib [71].

One initial approach to managing lenalidomide-associated fatigue is to suggest that patients take the lenalidomide dose in the evening. Other common causes of fatigue—such as anemia, infection, hypothyroidism, and, in patients receiving Rd, dexamethasone-related myopathy—should be considered, investigated, and treated if appropriate. If dexamethasone-related myopathy is suspected, the dexamethasone dose may be reduced. If fatigue is severe, lenalidomide dose reduction may be considered. Depression, anxiety, disturbed sleep, and reduced exercise capacity are also known to contribute to fatigue in patients with NDMM [70], and effective management of any such contributing factors can reduce symptoms. General supportive care includes individualized, moderate prescriptive exercise; pain relief (although this may exacerbate fatigue); treatment for depression; and measures to improve sleep [65, 70, 72].

### Infections

Although susceptibility to infections is increased in all patients with MM, elevated infection rates have been reported with Rd versus MPT in ASCT-ineligible NDMM patients, and with lenalidomide maintenance versus placebo post-ASCT (Tables 3 and 4) [21, 24, 29, 30, 73]. Meta-analyses indicate that the overall incidence of grade 3/4 or high-grade infection in lenalidomide-treated patients is around 14% [69, 74], including a number of fatal events in clinical trials [74]. The risk of infection appears to be greater during induction than during maintenance treatment [73]. Although the precise immunomodulatory effects of lenalidomide are still being elucidated, studies suggest that the drug has both immunostimulatory and immunosuppressive effects [34, 75]; it is likely that the latter, as well as the hematologic toxicity, play a role in the increased susceptibility to infection observed in lenalidomide-treated patients.

Preventive and therapeutic management of infection is essential for all patients with MM who receive lenalidomide treatment [73]. When lenalidomide is administered in combination with dexamethasone, routine antibiotic prophylaxis should be considered for the first 3 months of therapy—particularly in patients with aggressive disease, a history of infectious complications, or neutropenia [64]. Additionally, patients experiencing chronic non-neutropenic infections may require dexamethasone dose reductions. All patients receiving lenalidomide should be advised to seek medical attention promptly at the first sign of infection (e.g., cough, fever) [21]. In particular, patients with known risk factors for infection require close monitoring. As fatal outcomes have been reported following hepatitis B virus (HBV) reactivation, HBV status should be established before starting lenalidomide treatment, and patients with prior HBV infection must be closely monitored during therapy for signs of active HBV. Clinical data suggest that routine acyclovir prophylaxis in lenalidomide-treated patients can prevent varicella zoster/complicated herpes simplex virus infection, and should be considered in all patients receiving lenalidomide [76].

Vaccination may also be advisable to reduce the risk of infection. Studies of lenalidomide maintenance post-ASCT have reported responses to a number of common vaccines, including inactivated pertussis, diphtheria, tetanus, *Haemophilus influenzae*, and pneumococcal [77], as well as the live attenuated measles-mumps-rubella and herpes zoster [78], in up to 76% of patients, with no apparent vaccine-related side effects.

### Second primary malignancies

A number of studies and a comprehensive meta-analysis have shown that the risk of SPM development is increased in patients receiving lenalidomide versus control groups (Tables 3 and 4) [23, 29, 30, 79]. In a meta-analysis of seven clinical studies, including both first-line and maintenance treatments, the cumulative incidence of all SPMs at 5 years was 6.9% in NDMM patients who received lenalidomide versus 4.8% in those who did not (HR 1.55 [95% CI, 1.03–2.34]; *p* = 0.037) [79]. The observed difference appeared attributable primarily to an increased incidence of hematologic SPMs, the cumulative 5-year incidence of which was 3.1% in lenalidomide-treated patients versus 1.4% in controls (HR 3.8 [95% CI, 1.15–12.62]; *p* = 0.029), with no significant differences being observed in the rates of solid SPMs. Further, the authors concluded that the increased risk of hematologic SPMs was driven largely by treatment strategies incorporating both lenalidomide and melphalan [79]. In the FIRST trial, the incidence of SPMs was slightly reduced in the Rd versus the MPT arm (7% versus 9%) [24], and it should be noted that adding bortezomib to the Rd doublet does not appear to increase the SPM risk [16].

Studies suggest that lenalidomide maintenance therapy is associated with a marked increase in SPM risk; however, it should be noted that all patients who received post-ASCT lenalidomide maintenance in these studies had previously received high-dose melphalan, a known risk factor for SPMs [80]. Nevertheless, in the meta-analysis of data from three maintenance studies, rates of hematologic and solid-tumor SPMs were 6.1% and 7.3% among lenalidomide recipients versus 2.8% and 4.2% among patients assigned to placebo or observation [33]. Similar figures were reported for overall SPM incidence in patients receiving post-transplant maintenance with lenalidomide versus active observation in the Myeloma XI study (5.8% versus 2.0%; adjusted HR 1.65 [95% CI, 0.46–6.00]; *p* = 0.444) [81].

Despite the elevated risk of SPMs, the risk of death from MM significantly outweighs the risk of death from SPMs in patients treated with melphalan and either concurrent or sequential lenalidomide [82], and in those treated with lenalidomide maintenance [30, 33]. Nevertheless, clinicians should discuss the potential increased risk of SPMs with patients, to enable them to reach an informed decision. The IMWG consensus on SPMs recommends bone marrow examination with cytogenetic analysis for all patients initiating lenalidomide maintenance, and advocates “a low threshold for careful bone marrow analysis with karyotyping for patients with unexplained cytopenias that persist despite lenalidomide withdrawal” [82]. Further, consideration should be given to combining lenalidomide with agents other than melphalan [79], particularly in the light of data suggesting that, in patients ineligible for ASCT, Rd until progression is associated with a significant survival advantage relative to other first-line treatments incorporating melphalan (bortezomib, melphalan, and prednisone [VMP]; MPT; and MP) [83].

## Impact of long-term lenalidomide therapy on subsequent treatment

While the administration of lenalidomide as either first-line treatment or post-ASCT maintenance therapy can result in prolonged disease control, the majority of patients eventually relapse and require second-line therapy. At this point, prior lenalidomide treatment becomes a factor when selecting from the currently available therapeutic options. Several clinical issues are relevant, such as whether patients with biochemical relapse following long-term treatment with first-line Rd are truly refractory to lenalidomide, or could benefit from a lenalidomide-based second-line regimen. Further research is needed to answer this important question. In the maintenance setting, patients with biochemical relapse may benefit from treatment re-intensification (increasing lenalidomide to “full dose”/adding dexamethasone/adding a third compound), to prevent further and symptomatic disease progression.

A full, symptomatic relapse probably indicates that patients are refractory to lenalidomide, and second-line treatment will need to include agents with alternative modes of action, such as proteasome inhibitors, conventional chemotherapy, and/or monoclonal antibodies. Many of the new or emerging standards of care for second-line treatment are Rd-based combinations (e.g., carfilzomib-Rd [KRd], ixazomib-Rd [IRd], elotuzumab-Rd, daratumumab-Rd), although clinical trial data regarding their efficacy and safety in patients previously treated with lenalidomide are currently lacking; however, a retrospective analysis suggests that KRd may be effective in this setting [84]. Treatment re-intensification by combining lenalidomide with conventional cytotoxic agents is another second-line option that has shown promise [85–87]. In the phase 1/2 REPEAT trial, in which patients with lenalidomide-refractory MM received lenalidomide, low-dose cyclophosphamide, and prednisone until disease progression, 67% of patients achieved a partial response or better, including 23% VGPR and 5% CR [85]. Median PFS and OS after 24.5 months of follow-up were 12.1 and 29.0 months, respectively. Notably, response and survival were similar in patients with high- versus standard-risk cytogenetics, and the hematologic toxicity of this regimen was similar to that seen with Rd.

In the absence of strong clinical trial evidence, decisions regarding second-line therapy in patients who have become refractory to lenalidomide must be made on a case-by-case basis, taking into account factors such as the duration of first-line lenalidomide treatment, prior responsiveness to lenalidomide, previous and current toxicities, and the patient’s condition and risk status [88]. A number of alternatives to Rd-based combinations—such as pomalidomide, bortezomib, and dexamethasone (PVd) [89] and elotuzumab, pomalidomide, and dexamethasone (EPd) [90]—are now available, with more under investigation. Long-term toxicities may also influence subsequent treatment selection. Patients with renal complications should avoid nephrotoxic drugs where possible [72] and, although neuropathy is uncommon with lenalidomide, neurotoxic agents should be avoided in patients with long-lasting neurological symptoms. In transplant-ineligible patients relapsing more than 6 months after discontinuing lenalidomide, repeating first-line Rd treatment is appropriate [15].

## Conclusions

A substantial body of research has established lenalidomide as a key therapeutic option for patients with NDMM, both as a component of first-line regimens for patients ineligible for ASCT and as maintenance therapy post-ASCT. A number of studies in which newer therapies (e.g., ixazomib, elotuzumab) have been added to the Rd backbone are currently underway in ASCT-ineligible patients; their results are eagerly anticipated. The finding that adding daratumumab to Rd deepens response and significantly prolongs survival versus Rd alone highlights the significant benefits that can be achieved by combining Rd with novel agents [25]. In the maintenance setting, combinations of other agents with lenalidomide are being investigated, and the outcomes of these lines of research are likely to further expand the clinical utility of lenalidomide. Consequently, it is anticipated that lenalidomide will continue to play an important role in the treatment of NDMM for some time to come, and physicians should be aware of optimal management approaches to achieve the best outcomes for their patients when using this drug.
